# Silibinin Inhibits ICAM-1 Expression via Regulation of N-Linked and O-Linked Glycosylation in ARPE-19 Cells

**DOI:** 10.1155/2014/701395

**Published:** 2014-06-17

**Authors:** Yi-Hao Chen, Ching-Long Chen, Chang-Min Liang, Jy-Been Liang, Ming-Cheng Tai, Yun-Hsiang Chang, Da-Wen Lu, Jiann-Torng Chen

**Affiliations:** ^1^Graduate Institute of Medical Science, National Defense Medical Center, No. 161, Section 6, Minquan East Road, Taipei 114, Taiwan; ^2^Department of Ophthalmology, Tri-Service General Hospital, National Defense Medical Center, No. 325, Section 2, Cheng-Kong Road, Taipei 114, Taiwan; ^3^Graduate Institute of Aerospace and Undersea Medicine, National Defense Medical Center, No. 161, Section 6, Minquan East Road, Taipei 114, Taiwan

## Abstract

To evaluate the effects of silibinin on intercellular adhesion molecule-1 (ICAM-1) expression, we used ARPE-19 cells as a model in which tumor necrosis factor (TNF-*α*) and interferon (IFN-*γ*) enhanced ICAM-1 expression. This upregulation was inhibited by silibinin. In an adherence assay using ARPE-19 and THP-1 cells, silibinin inhibited the cell adhesion function of ICAM-1. The inhibitory effects of silibinin on ICAM-1 expression were mediated via the blockage of nuclear translocation of p65 proteins in TNF-*α* and phosphorylation of STAT1 in IFN-*γ*-stimulated cells. In addition, silibinin altered the degree of N-linked glycosylation posttranslationally in ARPE-19 cells by significantly enhancing *MGAT3* gene expression. Silibinin can increase the O-GlcNAc levels of glycoproteins in ARPE-19 cells. In a reporter gene assay, PUGNAc, which can also increase O-GlcNAc levels, inhibited NF-*κ*B reporter activity in TNF-*α*-induced ARPE-19 cells and this process was augmented by silibinin treatment. Overexpression of *OGT* gene was associated with reduced TNF-*α*-induced ICAM-1 levels, which is consistent with that induced by silibinin treatment. Taken together, silibinin inhibits ICAM-1 expression and its function through altered O-linked glycosylation in NF-*κ*B and STAT1 signaling pathways and decreases the N-linked glycosylation of ICAM-1 transmembrane protein in proinflammatory cytokine-stimulated ARPE-19 cells.

## 1. Introduction

Uveitis is an ocular inflammatory disease with multiple causes, such as infection or autoimmune reactions, and is characterized by a breakdown of the ocular blood barrier [1]. This barrier is composed of retinal pigment epithelial cells (RPEs) and the endothelium of retinal vessels. During intraocular inflammation, RPE cells are also the target of a cytokine network, in which tumor necrosis factor-*α* (TNF-*α*) and interferon-*γ* (IFN-*γ*) play central roles [2]. Cytokines are involved in inflammatory disorders, in which the expression of intercellular adhesion molecule-1 (ICAM-1) is induced in RPE cells [3]. ICAM-1 is a transmembrane glycoprotein with a variable degree of glycosylation and is a fundamental component in many immune-related processes [4]. The major functions of ICAM-1 are T-cell activation and leukocyte-endothelial cell interaction via lymphocyte function-associated antigen-1 and macrophage-1 antigen targeting [5]. ICAM-1 is also known to be involved in experimental models of uveitis [6]. In addition to uveitis, proliferative vitreoretinopathy, diabetic retinopathy, and macular pucker are all associated with ICAM-1 expression [7–9]. Thus, the control of ICAM-1 expression may lead to therapy for these diseases.

Silibinin is the primary component of the silymarin complex extracted from milk thistle. Silibinin has been shown to have antioxidant effects, estrogenic activity, and drug transporter (P-glycoprotein) modulatory function [10]; it has also been shown to have specific effects on gene expression via the suppression of NF-*κ*B transcription factor activity [11], as well as cardioprotective, neuroprotective, and hepatoprotective activities [12]. It was recently shown that silibinin exerts its anticancer effects via multiple molecular mechanisms that could block all stages of carcinogenesis, including initiation, promotion, and progression, and its possible usefulness as a preventive and therapeutic agent in cancer therapy has been authenticated [13]. Silymarin has been reported to decrease TNF-*α*-induced ICAM-1 expression in human umbilical vein endothelial cells [14]. In addition, according to our previous studies, ICAM-1 expression induced by proinflammatory cytokines may play a role in ocular inflammatory diseases [15, 16]; therefore, downregulation of ICAM-1 expression may be a therapeutic strategy for ocular inflammatory diseases. However, the effects of silibinin on ocular inflammatory models remain unknown.

In this study, we evaluated the effects of silibinin using an* in vitro* ocular cell model and found that silibinin inhibited mature ICAM-1 expression and its related functions in proinflammatory cytokine-stimulated ARPE-19 cells. The inhibitory effects of silibinin on ICAM-1 expression were mediated through blockage of the NF-*κ*B and STAT1 signal transduction pathways. In addition, we found that silibinin exerted its effects via modulation of N-linked glycosylation on the ICAM-1 protein itself and O-linked glycosylation on proteins of the NF-*κ*B and STAT1 signal transduction pathways. Our study provides insights into the effects of silibinin on ocular inflammation and elucidates a possible new mechanism of action for silibinin.

## 2. Materials and Methods

### 2.1. Cells

ARPE-19 cells were obtained from the American Type Culture Collection (Manassas, VA, USA). These cells were maintained in Dulbecco's modified Eagle's medium (F-12) supplemented with 4 mM L-glutamine, 10% fetal bovine serum (FBS; Gibco, USA), 100 U/mL penicillin, and 100 mg/mL streptomycin at 37°C in 5% CO_2_ in air. The culture medium was replaced twice weekly. THP-1 human monocytic cells were maintained in RPMI 1640 medium supplemented with 10% FBS, penicillin G (100 U/mL), streptomycin (100 g/mL), and L-glutamine (2 mM).

### 2.2. Cell Viability Assay

The cell viability assay was based on the ready-to-use cell viability reagent 4-[3-(4iodophenyl)-2-(4-nitrophenyl)-2H-5-tetrazolio]-1,3-benzene disulfonate (WST-1; Roche Diagnostics, Indianapolis, IN, USA). After treatment for 24 h with various concentrations of silibinin (S0417, Sigma-Aldrich, USA) in serum-free medium, 10 *μ*L of the WST-1 reagent was added to the medium in each well. The cells were incubated in a humidified atmosphere at 37°C in 5% CO_2_/95% air for 1 h, the multititer plate was shaken thoroughly for 1 min, and the absorbance values were read at 450 nm. The background absorbance was measured in wells containing only the dye solution and culture medium. Cell viability data were obtained from at least three experiments with at least six wells at each concentration in separate 96-well plates. The mean optical density values corresponding to the untreated controls were set at 100%. The results are expressed as the percentage of the optical density of treated cells relative to that of untreated controls.

### 2.3. Western Blot Analysis

For western blot analysis, confluent cultured cells were preincubated with or without 100 *μ*M O-(2-acetamido-2-deoxy-D-glucopyranosylidene) amino-N-phenylcarbamate (PUGNAc; A7229, Sigma-Aldrich, USA) for 1 h or 2 *μ*g/mL tunicamycin (T7765, Sigma-Aldrich) for 18 h. The cells were then further incubated with or without 25, 50, or 100 *μ*M silibinin for 24 h before stimulation with TNF-*α* (10 or 20 ng/mL; Peprotech, USA) or IFN-*γ* (500 U/mL; Peprotech) for 24 h. Treated and untreated cells were washed with PBS, harvested by scraping, and centrifuged at 1,000 ×g. Cell pellets were resuspended and sonicated in cold lysis buffer (PRO-PREPTM Protein Extraction Solution; iNtRON Biotechnology, Korea). The lysates were centrifuged at 12,000 ×g for 10 min, and the protein concentration in the clear supernatant was determined by the BCA protein assay kit (Pierce, Rockford, IL, USA). In order to inhibit N-glycosylation by silibinin, the lysates (20 *μ*g) were further incubated with or without 500 mU peptide-N-glycosidase F (PNGase F) for 18 h. After that, the lysates were resolved by 10% SDS-PAGE and transferred to PVDF membranes. The membranes were blocked with 5% (w/v) milk for 1 h at room temperature and subsequently incubated for 1 h at room temperature with a 1 : 1,000 dilution of antibodies against GAPDH (Rockland, USA), *α*-tubulin (Santa Cruz Biotechnology, USA), ICAM-1 (Santa Cruz Biotechnology), NF-*κ*B subunit p65 (Santa Cruz Biotechnology), lamin B1 (Abcam, UK), phosphorylated I*κ*B (Cell Signaling, USA), phosphorylated STAT1 (Santa Cruz Biotechnology), total STAT1 (Santa Cruz Biotechnology), or O-GlcNAc (CTD110.6; Covance, Berkeley, CA, USA). The membranes were washed and incubated with horseradish peroxidase-conjugated secondary antibody (1 : 1,000; Jackson ImmunoResearch Laboratories, USA) for 1 h at room temperature, and the proteins were visualized using an enhanced chemiluminescence procedure (enhanced chemiluminescence reagent, Millipore, Billerica, MA, USA).

### 2.4. Cell Adhesion Assays

Adherence of the THP-1 cells to the ARPE-19 cells was assayed using a cell-cell adhesion assay, as described previously [17]. In brief, the THP-1 cells were cultured at a density of 6.0 × 10^4^ cells in 25 mmol/L HEPES-buffered M199 containing 10% FBS. The ARPE-19 cells were pretreated with inhibitors for 24 h. After silibinin pretreatment (25 and 50 *μ*M), the ARPE-19 cells were exposed to 10 ng/mL TNF-*α* or 500 U/mL IFN-*γ* and were then washed three times with PBS before performing the cell-cell adhesion assay. The THP-1 cells were labeled for 30 min with 5 *μ*mol/L calcein-AM (Molecular Probes, Inc., USA). The labeled THP-1 cells (5.0 × 10^5^) were cocultured with the ARPE-19 cells for 1 h, and the cocultured cells were then washed three times with PBS before obtaining fluorescence images at 485 nm excitation and 538 nm emission wavelengths using a SPOT II digital camera attached to a fluorescence microscope with Spot II data acquisition software (Diagnostic Instruments). For adhesion quantification, the calcein-AM fluorescence intensity was measured at 485 nm excitation and 538 nm emission wavelengths using a Fluoroskan ELISA plate reader (Labsystems Ltd. Oy, Vantaa, Finland).

### 2.5. Lectin-Binding Analysis

Phytohemagglutinin-L (L-PHA) is a plant lectin that specifically binds *β*-1,6-GlcNAc-branched N-glycans [18]. Flow cytometric measurement of L-PHA binding to the ARPE-19 cells was used to characterize the branching of N-linked oligosaccharides on surface proteins. Cells with increased metabolic flux through the hexosamine pathway (i.e., cells treated with 30 mM GlcNAc) were used as a positive control. The ARPE-19 cells were cultured to 80% confluence. The medium was then changed, and the cells were cultured in the presence of 30 mM GlcNAc or 50, 100, or 200 *μ*M silibinin in a medium containing 10% FBS for a further 24 h. The cells were then harvested with PBS-based enzyme-free dissociation buffer (Invitrogen-Gibco, Grand Island, NY, USA). After centrifugation, the cell pellets were rinsed, resuspended in PBS, and incubated with FITC-L-PHA (10 *μ*g/mL; Vector, Burlingame, CA, USA) and 1% BSA in PBS on ice for 15 min. After the cells had been washed once with four volumes of 1% BSA/PBS, they were analyzed on a FACScan flow cytometer.

### 2.6. RNA Isolation and Quantitative RT-PCR

Expression of the ICAM-1 gene in ARPE-19 cells was investigated at the mRNA level by RT-PCR. Total RNA from ARPE-19 cells was isolated according to the manufacturer's instructions (TRIzol Reagent; Invitrogen-Gibco). Oligonucleotide primers complementary to the 5′ and 3′ ends of ICAM-1 and GAPDH cDNAs were used in RT-PCR. The sequences of the oligonucleotide primers used to amplify ICAM-1 and GAPDH cDNAs were ICAM-1 forward 5′-CCGGAAGGTGTATGAACTG-3′ and reverse 5′-CAGTTCATACACCTTCCGG-3′ and GAPDH forward 5′-GCAGGGGGGAGCCAAAAGGG-3′ and reverse 5′-TGCCAGCCCCAGCGTCAAAG-3′. Oligo (dT)_12–18_ (1 mg)-primed total RNA (5 mg) was reverse transcribed using reverse transcriptase (SuperScript III RNase H; Invitrogen-Gibco) supplied with a first-strand synthesis system for RT-PCR (Invitrogen-Gibco). PCR reactions contained 2 *μ*L cDNA, 67 mM Tris-HCl (pH 8.8), 16 mM (NH_4_)_2_SO_4_, 1.5 mM MgCl_2_, 0.2 mM dNTPs, 10 pM sense primer, 10 pM antisense primer, and 1.25 U* Taq* DNA polymerase (Invitrogen-Gibco) in a 50 *μ*L reaction volume. Annealing temperatures and MgCl_2_ concentrations were optimized to create a one-peak melting curve. PCR reaction parameters were as follows: denaturation at 94°C for 3 minutes followed by 30 cycles of denaturation at 94°C for 30 seconds, annealing at 58°C for 30 seconds, and extension at 72°C for 30 seconds. PCR amplification of GAPDH was routinely used as a control to assess the integrity of the RNA and cDNA. Amplification reaction products (10 *μ*L) were resolved on 1.2% Tris-borate-EDTA- (TBE-) buffered agarose gels and visualized with ethidium bromide staining. To verify their authenticity, amplicons were excised from the gel, repurified, and subjected to DNA sequence analysis.

The real-time RT-PCR reaction of* MGAT3* was performed using the TaqMan method. The amplification reactions were made in duplicate, using 96-well plates with 0.5 *μ*L of primers + probe, 5 *μ*L of master mix, 2.5 *μ*L of cDNA, and 2.0 *μ*L of diethylpyrocarbonate-treated water. The thermal cycling profile consisted of an initial temperature of 50°C for 2 min, followed by a denaturation step at 95°C for 10 min and then 40 successive cycles at 95°C for 15 s and 60°C for 1 min. The TaqMan assay used in* MGAT3* was Hs02379589_s1 (Applied Biosystems, USA). The amount of target mRNA relative to the endogenous control expression and relative to values from the control group was calculated using the 2^−ΔΔCt^ method. mRNA expression levels were normalized using the expression of GAPDH as the endogenous housekeeping gene.

### 2.7. Reporter Gene Assay

The ARPE-19 cells (3 × 10^4^/well) were plated and maintained in the DMEM/F-12 medium with 10% FBS in 24-well dishes for 24 h. To measure the NF-*κ*B activity, the ARPE-19 cells were cotransfected with pCMV-luciferase (Promega, Milan, Italy) and either pTAL-secreted alkaline phosphatase (SEAP) or pNF-*κ*B-SEAP (Clontech, San Jose, CA, USA) at a ratio of 1 : 4 for 4 h using the polycationic detergent Lipofectamine Plus (Invitrogen-Gibco) according to the manufacturer's instructions. The ARPE-19 cells were maintained for 20 h and subsequently preincubated with or without 100 *μ*M PUGNAc for 1 h. The cells were further incubated with or without 50 *μ*M silibinin for 24 h before stimulation with TNF-*α* (20 ng/mL) for 24 h at 37°C. For each treatment, the experiments were performed in triplicate. The SEAP activity was determined in the culture supernatants, and the luciferase activity was measured in the cell lysates to normalize the transfection efficiency. The luciferase activity was assessed with the Promega Dual-Luciferase Reporter 1000 Assay System.

### 2.8. O-GlcNAc Transferase (OGT) Overexpression

pcDNA3.1-OGT and pcDNA3.1 were purchased from Open Biosystems (Huntsville, AL, USA) and Invitrogen Life Technologies (Carlsbad, CA, USA), respectively. ARPE-19 cells were transfected with the pcDNA3.1 and pcDNA3.1-OGT vectors according to the manufacturers' protocols. After incubation for 24 h, the transfected cells were harvested. OGT expression was confirmed by western blot analysis (Figure 8).

### 2.9. Statistical Methods

Normally distributed continuous variables were compared by one-way analysis of variance. When a significant difference between the groups was apparent, multiple comparisons of the means were performed with the Student-Newman-Keuls procedure. The data are presented as the means ± standard error. Each result is representative of at least three independent experiments. All statistical assessments were two-sided and evaluated at the 0.05 level of significance.

## 3. Results

### 3.1. Cytotoxicity of Silibinin to ARPE-19 Cells

**Figure 1:**
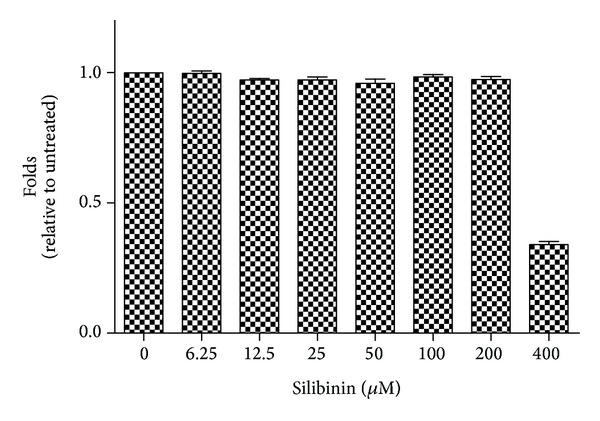
Inhibition of human retinal pigment epithelial cell line (ARPE-19) viability by silibinin. ARPE-19 cells were cultured for 24 h in the presence of different concentrations of silibinin, after which the cell viability was measured with a colorimetric test (WST-1). The results are expressed as mean fold of control.

The WST-1 assay was used to determine the cytotoxicity of silibinin to ARPE-19 cells, as shown in Figure 1. Limited cytotoxicity to ARPE-19 cells was noted for concentrations of silibinin lower than 200 *μ*M. Thus, 25, 50, or 100 *μ*M silibinin was used throughout the study.

### 3.2. Effects of Silibinin on ICAM-1 Expression Stimulated by TNF-*α* and IFN-*γ*

**Figure 2:**
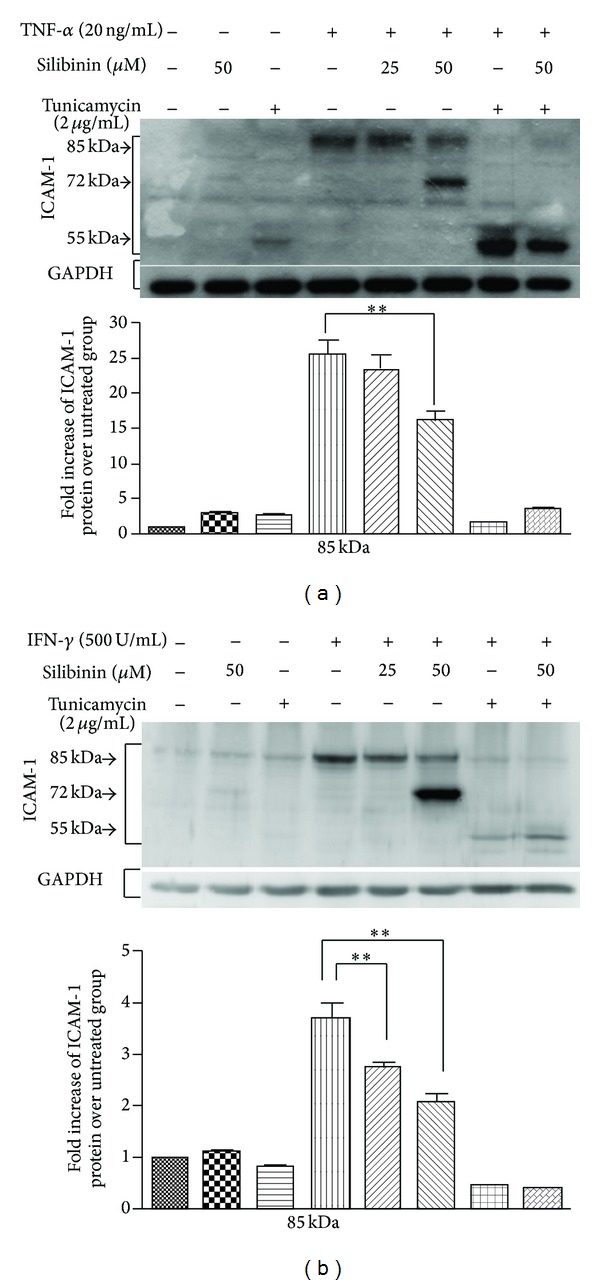
Silibinin suppresses TNF-*α*- or IFN-*γ*-induced ICAM-1 protein production in ARPE-19 cells. ARPE-19 cells were cultured to 80% confluence, treated with 25 *μ*M silibinin, 50 *μ*M silibinin, and/or 2 *μ*g/mL tunicamycin in a serum-free medium for 24 h, and stimulated with TNF-*α* (a) or IFN-*γ* (b) for a further 24 h. The levels of protein expression were quantified by western blotting using an antibody against ICAM-1 and were expressed as ratios relative to the average values for the 85 kDa protein in the control group. Asterisks (∗∗) designate responses that are significantly different (*P* < 0.01).

To evaluate the effect of silibinin on ICAM-1 expression in ARPE-19 cells, we treated ARPE-19 cells with TNF-*α* (Figure 2(a)) or IFN-*γ* (Figure 2(b)) in the presence or absence of silibinin. Stimulation of ARPE-19 cells with TNF-*α* or IFN-*γ* resulted in increased expression of mature ICAM-1, at a molecular weight of 85 kDa. Preincubation of the cells with 50 *μ*M silibinin diminished the TNF-*α*-dependent mature ICAM-1 expression, whereas the IFN-*γ*-dependent mature ICAM-1 expression was also decreased by treatment with 25 and 50 *μ*M silibinin.

### 3.3. Effects of Silibinin on Cell Adhesion Assays* In Vitro*

**Figure 3:**
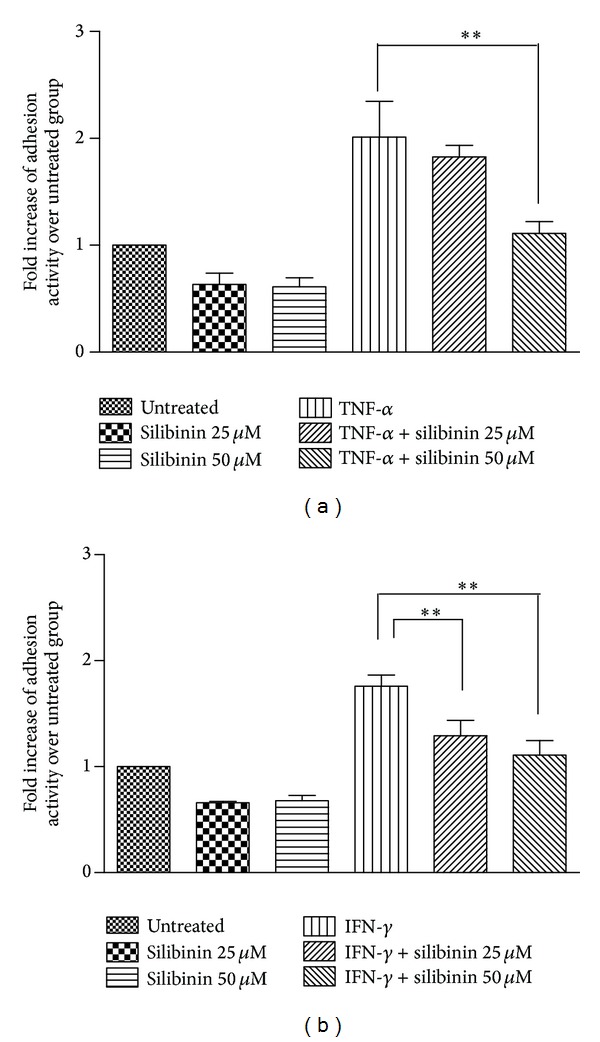
Decreased adhesion of THP-1 cells to ARPE-19 cells after inhibition of ICAM-1 production by silibinin. Confluent ARPE-19 cell cultures were preincubated with 25 *μ*M or 50 *μ*M silibinin for 24 h and subsequently stimulated with TNF-*α* (a) or IFN-*γ* (b) for 24 h. Fluorescein-labeled THP-1 cells were added to cytokine treated or untreated monolayers of ARPE-19 cells. The numbers of fluorescein-labeled THP-1 cells were determined. Data from five randomly selected low-power fields for each treatment are presented. Asterisks (∗∗) designate responses that are significantly different (*P* < 0.01).

To determine whether ICAM-1 function was associated with the silibinin-induced decrease in the levels of ICAM-1 expression, we next examined the effect of silibinin on TNF-*α*- or IFN-*γ*-induced monocyte adhesion (THP-1 cells) to ARPE-19 cells. TNF-*α* (Figure 3(a)) and IFN-*γ* (Figure 3(b)) increased the ability of monocytes to adhere to ARPE-19 cells, and silibinin reversed this phenomenon in a dose-dependent manner: 50 *μ*M silibinin significantly decreased the TNF-*α*-induced cell adhesion, and both 25 and 50 *μ*M silibinins also significantly diminished the IFN-*γ*-induced cell adhesion. These results confirm the relationship between silibinin and TNF-*α*- or IFN-*γ*-induced monocyte adhesion to ARPE-19 cells.

### 3.4. Effects of Silibinin on N-Linked Glycosylation of ICAM-1

Interestingly, the expression of a smaller isoform of ICAM-1 with an apparent molecular weight of 72 kDa was found in the group of ARPE-19 cells treated with 50 *μ*M silibinin after stimulation with either TNF-*α* (Figure 2(a)) or IFN-*γ* (Figure 2(b)). One of the possible reasons for this finding was that silibinin may modulate the N-linked glycosylation of ICAM-1, yielding ICAM-1 molecules with different molecular weights. Tunicamycin is an inhibitor of protein N-glycosylation and reportedly inhibits ICAM-1 N-glycosylation [19], leading to the expression of glycosylated ICAM-1 with a molecular mass of 50–95 kDa [20]. To provide support for our hypothesis that the altered degree of glycosylation was responsible for the observed lower molecular weight of ICAM-1, tunicamycin was used as a positive control to evaluate the effect of silibinin treatment on ICAM-1 N-glycosylation. In cell lysates treated with tunicamycin alone, ICAM-1 was observed to have an apparently smaller molecular weight (approximately 55 kDa), with or without TNF-*α* or IFN-*γ* stimulation. Compared with tunicamycin treatment, silibinin induced the expression of ICAM-1 with an apparently smaller molecular weight (approximately 72 kDa), and both TNF-*α* and IFN-*γ* induced the expression of mature ICAM-1 (85 kDa) in the ARPE-19 cells. This suggests that silibinin and tunicamycin modulate the N-glycosylation of newly synthesized ICAM-1.

**Figure 4:**
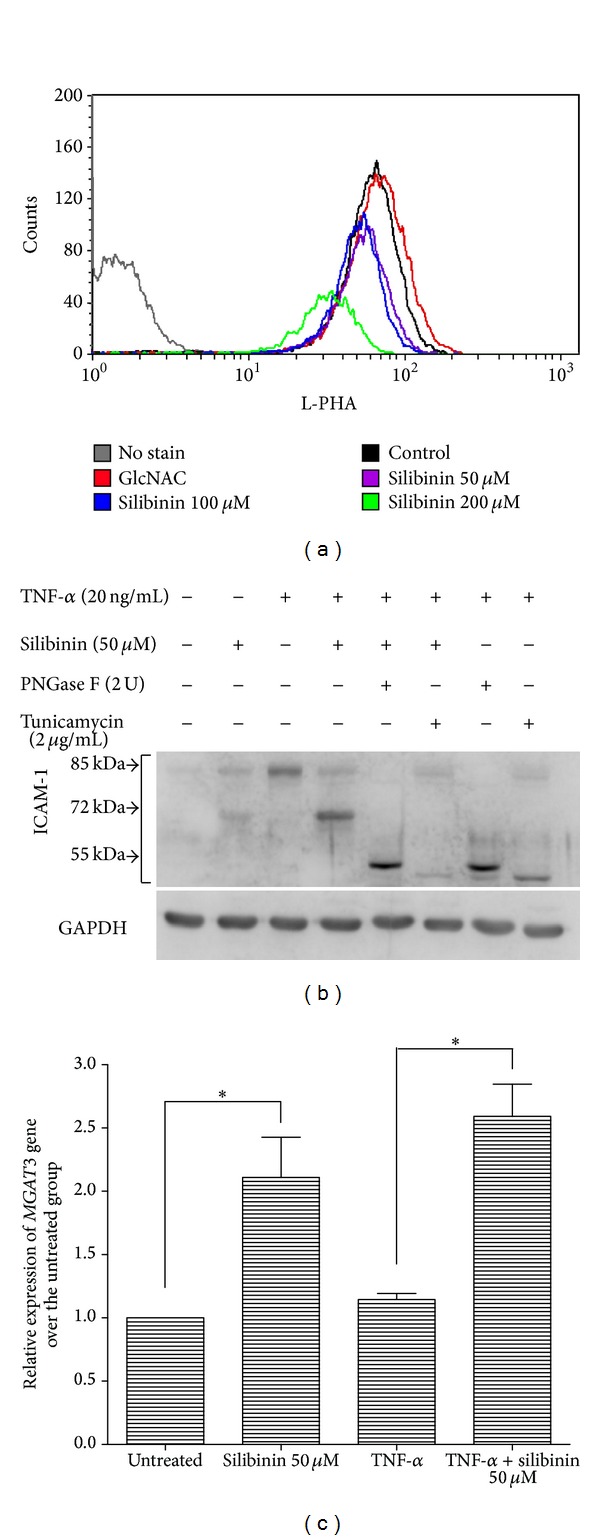
Evidence for inhibition of N-glycosylation by silibinin in ARPE-19 cells. (a) ARPE-19 cells at 80% confluence were treated with 30 mM GlcNAc, 25 *μ*M, or 50 *μ*M silibinin for 24 h. The cells were harvested and incubated with fluorescein isothiocyanate (FITC)-LPHA (10 *μ*g/mL), and L-PHA lectin binding was detected by flow cytometry. (b) The ARPE-19 cells were pretreated with 2 *μ*g/mL tunicamycin and/or 50 *μ*M silibinin, treated with or without TNF-*α*, and treated with or without PNGase F. The levels of protein expression were quantified by western blotting using an antibody against ICAM-1. (c) The ARPE-19 cells were pretreated with 50 *μ*M silibinin and then treated with or without TNF-*α*. The relative expression of* MGAT3* gene was detected by qRT-PCR. Asterisks (∗) designate responses that are significantly different at *P* < 0.05.

To confirm the hypothesis that silibinin modulates N-linked glycosylation in ARPE-19 cells, we assayed the levels of L-PHA binding on the surface of silibinin-treated ARPE-19 cells. Compared with the positive control cells, which had been treated with GlcNAc, cells cultured with silibinin for 24 h showed decreased binding of L-PHA. Reduction in L-PHA binding was observed in the cells treated with 50, 100, and 200 *μ*M silibinin compared with the positive and normal controls (Figure 4(a)). PNGase F is an amidase that cleaves between the innermost GlcNAc and asparagine residues of high mannose, hybrid, and complex oligosaccharides from N-linked glycoproteins [21]. To further prove that silibinin modulates N-linked glycosylation on ICAM-1 expression, we treated the expressed protein with or without PNGase F in TNF-*α*-stimulated ARPE-19 cells. The expression of ICAM-1 with a smaller apparent molecular mass of 72 kDa was induced by silibinin and that with 55 kDa was induced by tunicamycin in TNF-*α*-stimulated ARPE-19 cells. With the prolonged incubation of silibinin-treated cell lysates with PNGase F, the expression of the smaller molecular mass of ICAM-1 shifted from 72 kDa to 55 kDa, similar to the effects of tunicamycin. This proved that expression of the smaller isoform of ICAM-1 following silibinin treatment in TNF-*α*-stimulated ARPE-19 cells occurred through modulation of N-glycosylation on ICAM-1 (Figure 4(b)). To further evaluate the regulation of posttranslational glycosylation by silibinin, the activity of N-acetylglucosaminyltransferase III (GnT-III) encoded by the* MGAT3* gene was assessed via quantitative RT-PCR. Compared with the untreated group, expression of* MGAT3* mRNA significantly increased after silibinin treatment of ARPE-19 cells. Although expression of the* MGAT3* gene was unchanged after treatment of ARPE-19 cells with TNF-*α*, silibinin cotreatment significantly enhanced* MGAT3* gene expression (Figure 4(c)). Taken together, differences in the molecular weight of the nascent ICAM-1 may have occurred because of the modulation of the degree of N-glycosylation on ICAM-1 by silibinin via upregulation of the* MGAT3* gene.

### 3.5. Effects of Silibinin on ICAM-1 mRNA Induced by TNF-*α* or IFN-*γ*

**Figure 5:**
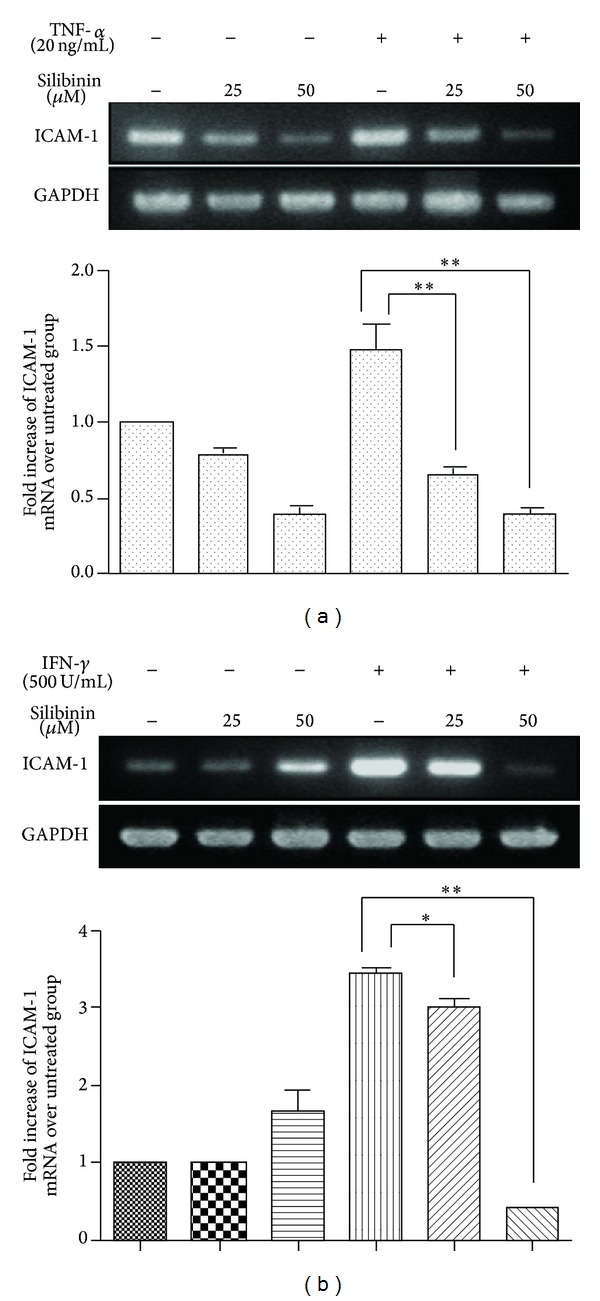
Effects of silibinin on gene expression of ICAM-1 in TNF-*α*- or IFN-*γ*-stimulated ARPE-19 cells. ARPE-19 cells were preincubated with 25 *μ*M or 50 *μ*M silibinin for 24 h and then stimulated with TNF-*α* (a) or IFN-*γ* (b) for 6 h. The levels of gene expression were quantified by RT-PCR and are expressed as ratios to the average of the control group. Asterisks (∗) and (∗∗) designate responses that are significantly different at *P* < 0.05 and *P* < 0.01, respectively.

To assess the effect of silibinin on mRNA levels of ICAM-1 induced by TNF-*α* or IFN-*γ*, we examined the mRNA of ICAM-1 in ARPE-19 cells by RT-PCR. Similar to the previous results [14], ICAM-1 was basally expressed at a very low level in ARPE-19 cells. Expression of ICAM-1 mRNA was significantly induced after treatment of ARPE-19 cells with either TNF-*α* or IFN-*γ* for 6 h. However, silibinin cotreatment significantly inhibited these effects (Figure 5).

### 3.6. Effects of Silibinin on TNF-*α*-Induced Translocation of the NF-*κ*B Subunit p65

**Figure 6:**
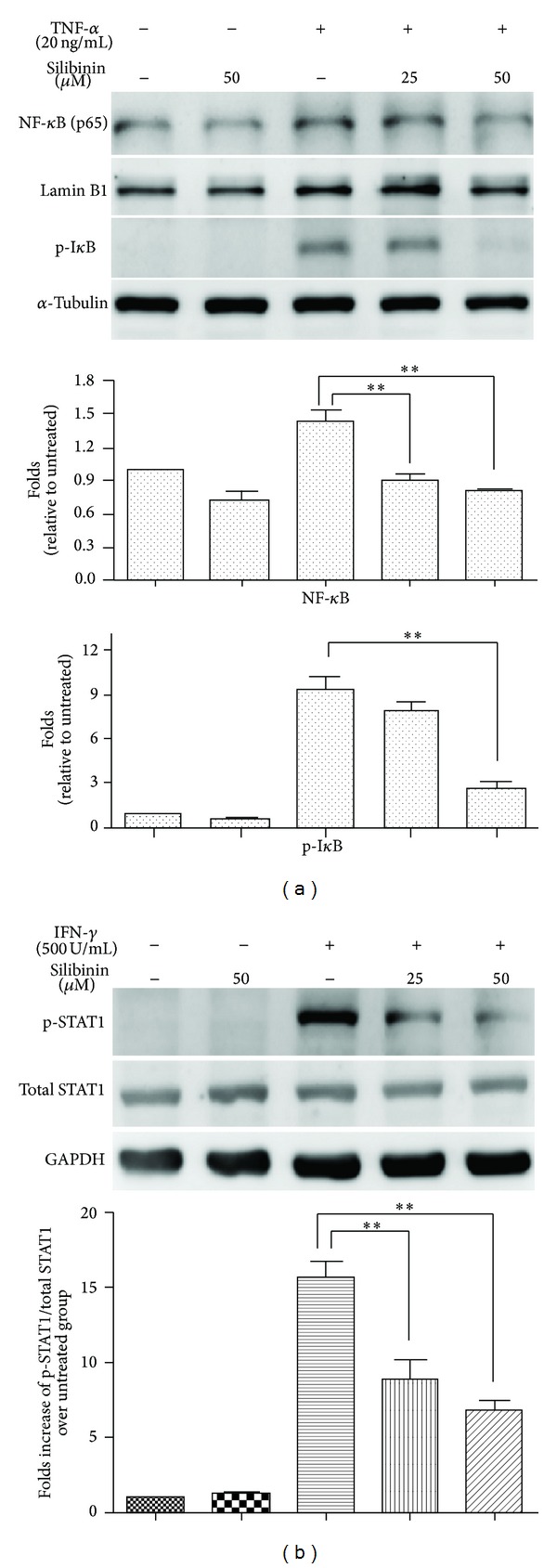
Inhibitory effects of silibinin on NF-*κ*B and STAT1 signaling pathways. ARPE-19 cells were pretreated with 25 *μ*M or 50 *μ*M silibinin under serum-free conditions for 24 h and incubated for a further 30 min in the presence or absence of TNF-*α* (a) or IFN-*γ* (b). Lysates of cells stimulated with TNF-*α* were blotted with an antibody against the p65 subunit of NF-*κ*B (nucleus) and an anti-phospho-I*κ*B antibody (cytoplasm). Lysates of cells stimulated with IFN-*γ* were blotted with anti-phospho- or anti-total STAT1 antibodies. Asterisks (∗) and (∗∗) designate responses that are significantly different at *P* < 0.05 and *P* < 0.01, respectively.

Because binding of the nuclear transcription factor NF-*κ*B to the enhancer is essential for activation of TNF-*α*-induced ICAM-1 expression [14, 15], we further studied whether silibinin could modify NF-*κ*B binding activity and in turn alter ICAM-1 expression in ARPE-19 cells. NF-*κ*B is composed of two groups of structurally related interacting proteins, one of which is the p65 subunit. When the signaling pathway is activated, NF-*κ*B translocates from the cytoplasm into the nucleus and binds to recognition sites on DNA, resulting in elevated levels of p65 in the nucleus [22]. Indeed, western blot analysis with anti-p65 antibodies revealed that treatment of ARPE-19 cells with TNF-*α* resulted in an increase of p65 levels in the nucleus. However, p65 translocation was inhibited by pretreatment of cells with silibinin in a dose-dependent manner (Figure 6(a)). This suggested that silibinin could inhibit TNF-*α*-induced translocation of NF-*κ*B into the nucleus.

### 3.7. Effects of Silibinin on TNF-*α*-Induced Phosphorylation of I*κ*B

I*κ*B is an inhibitory protein that prevents translocation of NF-*κ*B to the nucleus. However, after phosphorylation, I*κ*B is degraded, allowing NF-*κ*B to translocate into the nucleus [22]. Stimulation of the control cells with TNF-*α* resulted in enhanced phosphorylation of I*κ*B in the cytoplasm. Preincubation of the cells with silibinin in the medium diminished the TNF-*α*-dependent phosphorylation of I*κ*B in a dose-dependent manner (Figure 6(a)). This suggested that silibinin could inhibit phosphorylation of I*κ*B, thereby preventing further NF-*κ*B translocation after TNF-*α* stimulation.

### 3.8. Effects of Silibinin on IFN-*γ*-Mediated Phosphorylation of STAT1

IFN-*γ* induction of ICAM-1 transcription occurs through the activation of Janus kinases (JAKs) and activators of transcription (STAT) signal transduction pathways [15, 23]. When the signaling pathway is activated, STAT1 is phosphorylated and subsequently translocates into the nucleus [24]. We investigated whether silibinin modifies the phosphorylation of STAT1 in ARPE-19 cells that were stimulated with IFN-*γ*. Phosphorylated STAT1 was not detected in the control or cells treated with silibinin alone. STAT1 phosphorylation levels increased after treatment with IFN-*γ*. However, treatment with silibinin lowered the level of phosphorylated STAT1 in IFN-*γ*-activated cells (Figure 6(b)). This suggests that silibinin can inhibit the phosphorylation of STAT1 and thus prevent IFN-*γ*-mediated STAT1 activation.

### 3.9. Effect of Silibinin on O-GlcNAc Protein Levels in ARPE-19 Cells

**Figure 7:**
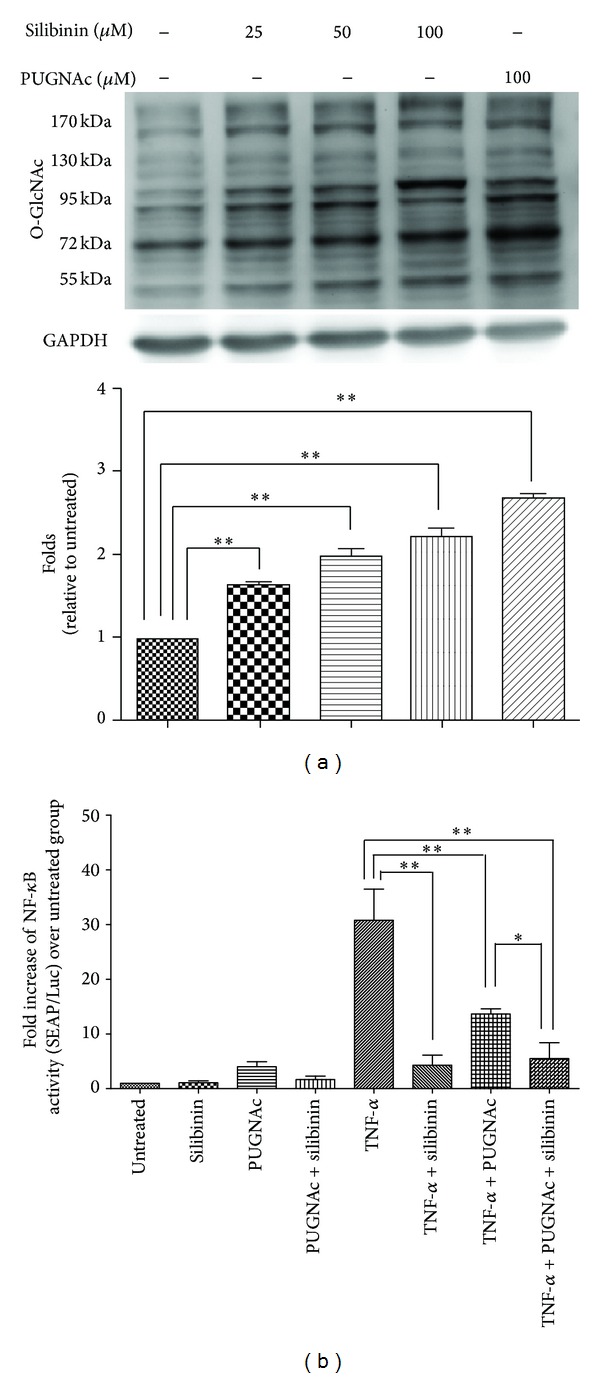
Effects of silibinin on O-GlcNAc protein levels and TNF-*α*-induced activation of the NF-*κ*B pathway. (a) ARPE-19 cells at 80% confluence were treated with 100 *μ*M PUGNAc for 1 h and 25, 50, or 100 *μ*M silibinin for 24 h. The blot was probed with an antibody against O-GlcNAc. The levels of protein expression were quantified by western blotting and are expressed as ratios relative to the average values for the control group. (b) The ARPE-19 cells were pretreated with 100 *μ*M PUGNAc and/or 50 *μ*M silibinin and were subsequently treated with or without TNF-*α*. A reporter gene assay was used to detect the activation of the NF-*κ*B pathway. Asterisks (∗) and (∗∗) designate responses that are significantly different at *P* < 0.05 and *P* < 0.01, respectively.

The activity of the NF-*κ*B signaling pathway has been associated with O-GlcNAc protein levels in previous studies, which showed that increased protein O-linked glycosylation attenuated NF-*κ*B activity [25, 26]. However, it was unclear whether the inhibitory effect of silibinin on NF-*κ*B activity was associated with O-GlcNAc protein levels. To evaluate the effects of silibinin on O-GlcNAc levels of glycoproteins in ARPE-19 cells, we treated ARPE-19 cells with silibinin and PUGNAc. Silibinin dose-dependently increased glycoprotein O-GlcNAc levels in the ARPE-19 cells, which was similar to the effect of PUGNAc, an inhibitor of N-acetylglucosaminidase (OGA) that increases O-GlcNAc levels (Figure 7(a)). This result suggests that silibinin can increase glycoprotein O-GlcNAc levels in ARPE-19 cells.

### 3.10. Relationship between the Attenuating Effect of Silibinin on the Activation of the NF-*κ*B Pathway and Increased O-GlcNAc Levels

As described previously, silibinin increased glycoprotein O-GlcNAc levels in ARPE-19 cells (Figure 7(a)) and inhibited ICAM-1 expression and synthesis by inhibiting NF-*κ*B activity in TNF-*α*-treated ARPE-19 cells (Figures 2(a) and 6(a)). This prompted us to investigate whether silibinin attenuates the TNF-*α*-induced activation of the NF-*κ*B pathway by increasing the O-GlcNAc levels in ARPE-19 cells using an NF-*κ*B reporter assay. We found that silibinin inhibited NF-*κ*B reporter activity in TNF-*α*-treated ARPE-19 cells. PUGNAc also inhibited NF-*κ*B reporter activity in TNF-*α*-induced ARPE-19 cells, and this process was augmented by silibinin (Figure 7(b)). This result suggests that silibinin attenuates NF-*κ*B signaling by increasing the O-GlcNAc levels in TNF-*α*-treated ARPE-19 cells.

### 3.11. Comparison between the Effects of Silibinin Treatment and OGT Overexpression on TNF-*α*-Induced ICAM-1 Expression in ARPE-19 Cells

**Figure 8:**
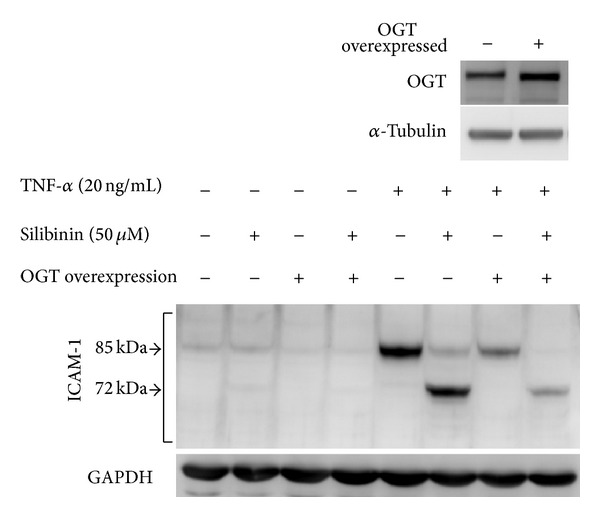
Comparison of the effects of GlcN and OGT overexpression on TNF-*α*-induced ICAM-1 expression. ARPE-19 cells were transfected with pcDNA3.1-OGT and/or pretreated with 50 *μ*M silibinin. The cells were then treated with TNF-*α* for 24 h in the absence or presence of silibinin or were transfected with pcDNA3.1-OGT. Whole-cell lysates were prepared and analyzed with western blots using antibodies directed against ICAM-1.

Silibinin increased glycoprotein O-GlcNAc levels in the ARPE-19 cells (Figure 7), which may mimic the effect of overexpression of OGT, an enzyme that increases O-GlcNAc levels. We further compared the effect of silibinin with that of OGT overexpression on TNF-*α*-induced ICAM-1 expression in ARPE-19 cells (Figure 8). Overexpression of OGT was associated with reduced TNF-*α*-induced ICAM-1 levels. This effect is consistent with that induced by silibinin treatment. Moreover, the reduced molecular mass of ICAM-1 was achieved only with silibinin treatment. This finding was also compatible with our previous findings (Figures 2 and 4), which demonstrated that silibinin influences N-linked glycosylation modifications of ICAM-1. These data suggest that silibinin altered both N-linked and O-linked glycosylations of ICAM-1, the expression of which is induced by TNF-*α* in ARPE-19 cells.

## 4. Discussion

Silibinin is a major component of traditional herbs and is widely used in the treatment of hepatic diseases. Recently, it has also been studied for its antitumor effects and it is a potential adjunctive agent for cancer therapies [27]. In addition to its anticancer effects, silibinin has been reported to have potent anti-inflammatory effects: it can inhibit the production of several proinflammatory cytokines, such as TNF-*α*, interleukin- (IL-) 1*β*, or IL-6 [28]. Moreover, silymarin has been reported to suppress TNF-*α*-induced ICAM-1 production in human umbilical vein endothelial cells [14]. In our previous studies, we have shown that proinflammatory cytokines also play important roles in ocular inflammatory diseases, which are related to ICAM-1 expression. In ocular diseases, silibinin has been demonstrated to have an inhibitory effect on rat lens aldose reductase and has the potential for reducing diabetic cataract formation [29]. However, the effect of silibinin on ocular inflammatory diseases remains unknown. In the present study, we evaluated the effects of silibinin on TNF-*α*- or IFN-*γ*-induced ICAM-1 expression in the ARPE-19 cell* in vitro*. We noted that silibinin suppressed TNF-*α*- or IFN-*γ*-induced production of mature ICAM-1 protein in ARPE-19 cells via downregulation of their related signaling pathways and further decreased ICAM-1-related functions such as cell adhesion ability. Furthermore, we reported, for the first time, that silibinin increased O-GlcNAc protein levels, attenuated NF-*κ*B reporter activity, and inhibited ICAM-1 expression in TNF-*α*-treated ARPE-19 cells. In addition, we found that silibinin reduced the N-glycosylation of ICAM-1, which in turn reduced its molecular weight.

The ICAM-1 protein is a transmembrane glycoprotein of 505 amino acids with a molecular mass that varies from 80 to 114 kDa, depending on the degree of glycosylation. Because of its heavy glycosylation at four sites where N-linked glycans can be attached [30], we were easily able to detect differences in expression before and after silibinin treatment. We observed that silibinin treatment altered the molecular mass of ICAM-1 from 85 to 72 kDa in ARPE-19 cells treated with either TNF-*α* or IFN-*γ*. Treatment with tunicamycin alone shifted the molecular mass of ICAM-1 to approximately 55 kDa. With silibinin and tunicamycin cotreatment, the molecular mass of ICAM-1 shifted from approximately 72 kDa to approximately 55 kDa. Compared with silibinin treatment, treatment with tunicamycin induced the formation of more hypoglycosylated ICAM-1. On the other hand, we also noted that the expression of L-PHA-binding oligosaccharides on the surface of the ARPE-19 cells was reduced after silibinin or tunicamycin treatment. These findings suggest that silibinin reduces the molecular mass of ICAM-1 by reducing its N-glycosylation. GnT-III catalyzes the transfer of GlcNAc to a core *β*-mannose residue in N-linked oligosaccharides, resulting in the formation of a bisected sugar chain [31]. This unique structure inhibits the action of GnT-V, which are involved in the formation of *β*-1,6-GlcNAc-branched sugar chains [32]. Thus, silibinin upregulated the expression of the* MGAT3* gene, resulting in the formation of bisected sugar chains, which reduced the expression of *β*-1,6-GlcNAc-branched sugar chains. This was compatible with the observed result of diminished L-PHA binding following silibinin treatment.

ICAM-1 expression is primarily regulated through the following four primary pathways: NF-*κ*B, JAK/STAT, AP-1, and PKC [23]. The NF-*κ*B pathway is activated by proinflammatory cytokines such as TNF-*α* and IL1-*β*. In the resting state, NF-*κ*B resides in the cytoplasm as a heterotrimer consisting of p50, p65, and the inhibitory subunit I*κ*B. When the pathway is activated, the I*κ*B protein is phosphorylated and subsequently degraded. In the absence of the inhibitory subunit, p50 and p65 are released and translocated to the nucleus to bind specific DNA sequences present in the promoters of various genes. It is known that silibinin is a powerful inhibitor of the activation of NF-*κ*B signaling pathways, which provides, in part, the molecular basis for its anticancer, antiapoptotic, and anti-inflammatory effects [33, 34]. We further found that silibinin can suppress the phosphorylation of I*κ*B and reduce the translocation of the p65 subunit of NF-*κ*B to the nucleus.

The JAK/STAT pathway is stimulated by IFN-*γ*. JAKs bind to some cell surface cytokine receptors, and the binding of the ligand to the receptor triggers the activation of JAKs, which phosphorylate tyrosine residues on the receptor. Proteins that contain phosphotyrosine-binding SH2 domains, such as STATs, are recruited to the receptors and are then themselves tyrosine-phosphorylated by JAKs. These phosphorylated STATs dimerize, resulting in their activation. Activated STAT dimers accumulate in the cell nucleus and in turn activate transcription of their target genes. Silymarin has been shown to have a preventive effect on rat brain injury [35] and an antiproliferative effect [36], both of which are mediated via STAT signaling pathways. We noted that silibinin can inhibit the formation of phosphorylated STAT1 and prevent its downstream activation of the gene encoding ICAM-1. Taken together, these findings indicate that TNF-*α*- or IFN-*γ*-induced upregulation of ICAM-1 in ARPE-19 cells is inhibited by silibinin through the related NF-*κ*B and STAT1 signaling pathways, respectively.

The transcription factor NF-*κ*B mediates various aspects of cell biology, such as cytokine production, lymphocyte activation, and cell proliferation [37, 38]. Activation of NF-*κ*B requires posttranslational modifications, including phosphorylation, acetylation, and glycosylation. Several previous studies have shown that enhancing O-GlcNAc modification of the NF-*κ*B subunit may promote expression of its related genes [39, 40]. O-GlcNAc modification of NF-*κ*B at Thr-352 in response to high glucose has been shown to inhibit the interaction of NF-*κ*B with I*κ*B, causing the nuclear translocation of NF-*κ*B and activation of its target genes [41]. However, contradictory findings have also been reported, where increased O-GlcNAc modification of NF-*κ*B at a different site may in contrast inhibit TNF-*α*-induced expression of inflammatory mediators through inhibition of NF-*κ*B p65 signaling. Treatments that inhibit TNF-*α*-induced NF-*κ*B p65 activation increase O-GlcNAc modification of NF-*κ*B p65 and inhibit NF-*κ*B p65 phosphorylation at serine 536, thus promoting I*κ*B binding to NF-*κ*B p65 [25, 42]. Our previous study also reported that increasing the levels of O-GlcNAc-modified protein with glucosamine attenuated the NF-*κ*B signaling pathway [26]. Consistent with these data, this study demonstrated that NF-*κ*B reporter activity was inhibited by silibinin, PUGNAc, or both, in TNF-*α*-induced ARPE-19 cells. O-GlcNAc protein levels are regulated by the activities of two key enzymes: OGT, which catalyzes the addition of O-GlcNAc, and OGA, a neutral hexosaminidase responsible for O-GlcNAc removal. PUGNAc is an inhibitor of OGA and is responsible for increasing O-GlcNAc protein levels [43], which is similar to the effect of silibinin and overexpression of OGT. Furthermore, we examined whether OGT overexpression mimicked the effects of silibinin with respect to attenuating TNF-*α*-induced ICAM-1 expression in ARPE-19 cells. Our results suggest that, similar to silibinin, OGT overexpression attenuates TNF-*α*-induced ICAM-1 expression in ARPE-19 cells. Taken together, these results support the hypotheses that silibinin treatment reduces TNF-*α*-induced ICAM-1 expression in ARPE-19 cells and that this effect is associated with an increase in O-GlcNAc protein levels.

Silibinin has been proposed to be beneficial for diabetes [44–46], and reports have demonstrated that treatment with silibinin or silymarin in patients with type 2 diabetes or diabetic rats could decrease the blood sugar concentration [47, 48]. Furthermore, silymarin may reduce the burden of advanced glycation endproducts (AGEs) in diabetes and diabetic complications [49]. However, the process of AGE formation is different from that of N-linked or O-linked glycosylation. N-linked or O-linked glycosylation is site-specific enzymatic process, whereas glycation in AGE formation is a nonenzymatic form of glycosylation. No previous studies have discussed the effect of silibinin on enzymatic glycosylation. Silybin and dehydrosilybin mediate the inhibition of cellular glucose uptake by directly interacting with GLUT transporters [50], and the inhibition of hepatic glucose-6-phosphatase and gluconeogenesis by silibinin [51] may explain why silibinin has been beneficial in the treatment of diabetes. These findings indicate that the glucose cycle in cells may be influenced by silibinin, which may be compatible with our novel observation that silibinin has effects on glycosylation. On the other hand, in the past, silibinin was reported to exert a wide range of functions, and the novel observations of our study may shed more light on this phenomenon.

In summary, we used silibinin to inhibit TNF-*α*- or IFN-*γ*-mediated mature ICAM-1 expression in ARPE-19 cells* in vitro*, which is a key event in the pathogenesis of uveitis. Silibinin also abrogated cell adhesion in cultured RPE cells. The downregulation of NF-*κ*B or STAT1 activities with silibinin decreased the production of ICAM-1. However, N-glycosylation of ICAM-1 was partially influenced by silibinin, and this may also diminish cell adhesion. In addition to its effect on N-glycosylation, silibinin was able to increase the overall O-GlcNAc protein levels, which decreased the activity of the NF-*κ*B reporter gene system. Given these findings, we conclude that silibinin inhibits proinflammatory cytokine-related signaling pathways through altered O-linked glycosylation in RPE cells and modifies the degree of N-linked glycosylation of downstream target proteins such as ICAM-1. Silibinin plays a key role in the treatment of ocular inflammatory diseases, and its effects on N-linked and O-linked glycosylation may indicate a further mechanism underlying its antioxidative, anticancer, and neuroprotective effects.
